# Prevalence of Herpes Simplex and Varicella-Zoster Virus DNA in Corneal Grafts Is Higher than Expected

**DOI:** 10.3390/microorganisms11102405

**Published:** 2023-09-26

**Authors:** Marie Ella Horstmann, Mohammad Al Hariri, Stephanie D. Grabitz, Julia Bing Bu, Melissa Apel, Norbert Pfeiffer, Joanna Wasielica-Poslednik

**Affiliations:** 1 Department of Ophthalmology, University Medical Center of the Johannes Gutenberg-University Mainz, 55131 Mainz, Germanyjoanna.wasielica-poslednik@unimedizin-mainz.de (J.W.-P.); 2 Eye Bank of Rhineland-Palatinate in Mainz, 55131 Mainz, Germany

**Keywords:** corneal graft, donor cornea, HSV-1, HSV-2, VZV, CMV, donor cornea, transplantation, PCR

## Abstract

Purpose: (1) To determine the prevalence of herpes simplex virus type 1 (HSV-1) and type 2 (HSV-2), varicella-zoster virus (VZV), and cytomegalovirus (CMV) DNA in donor corneas; (2) To evaluate the clinical outcome of the grafts with viral DNA and to compare donors with and without viral DNA. Methods: We analyzed data from all donors and recipients who underwent penetrating keratoplasty (PK) or Descemet membrane endothelial keratoplasty (DMEK) between September 2022 and March 2023. Donor corneoscleral rims and excised recipients’ corneal buttons were tested for the presence of HSV-1, HSV-2, VZV, and CMV DNA by polymerase chain reaction (PCR). The results were known 2–3 days after the surgery. We closely followed up on patients whose grafts tested positive for viral DNA. We compared the medical histories of donors with and without viral DNA. Results: We included 85 corneas from 67 donors. Seven (8.2%) donor corneas tested positive for HSV-1 (n = 3) or VZV (n = 4) DNA. We did not detect any HSV-2 or CMV DNA. In the postoperative follow-up of patients with positive PCR, a graft failure was observed in one and infections in two eyes. Re-operation was necessary in three of these cases (42.9%). Patients without herpes DNA in the donor cornea needed reoperation in 7.7% of the cases. Cultural duration, the cause of the donor’s death, and the death-to-explantation interval did not differ significantly between donors with and without viral DNA. Additionally, 3 of the 7 (42.9%) donors with positive PCR were in a septic status at the time of death, compared to 21 of the 78 (26.9%) donors with negative PCR (*p* = 0.52). Conclusions: The prevalence of herpes DNA in the donor corneas was 8.2% and thus higher than previously reported. We did not notice any evidence for a donor-to-host transmission, but a higher rate of postoperative complications in recipients of the grafts with viral DNA. The donors with and without herpetic DNA did not differ significantly regarding systemic diagnoses or cultural conditions, but sepsis was more frequent in the group with viral DNA.

## 1. Introduction

Herpes viruses infect the majority of the world’s human population. An estimated 67% of the population under the age of 50 is infected with herpes simplex virus type 1 (HSV-1). The virus contracts through the mucus membranes during childhood and remains latent in the ophthalmic division of the trigeminal ganglion. Its reactivation causes recurrent infections [1].

Approximately 99% of people over the age of 40 in Germany have faced a varicella-zoster virus (VZV) (human herpes virus 3) infection, and about 20–30% of people develop symptomatic VZV infection during their lives. It occurs primarily in the elderly and/or immunocompromised people. The lifetime prevalence is 25–50% [2].

Ocular symptoms of herpetic infection can present as blepharitis, conjunctivitis, epithelial keratitis, stromal keratitis, endotheliitis, iritis, trabeculitis, and retinitis [3,4]. The symptomatic as well as the asymptomatic infections of the cornea with herpes viruses may be detected with polymerase chain reaction (PCR).

Nowadays, all these clinical forms may be treated with antiviral local and systemic medications. However, recurrent and untreated herpes keratitis (HK) may cause corneal opacities and vascularization, resulting in severe visual impairment [3,4,5]. In such cases, only surgical treatment, e.g., corneal transplantation, can restore vision and relieve symptoms of the patients.

A history of HK in the operated eye is a high-risk factor for HK recurrence in the graft and graft failure [6]. Hence, in case of a preoperative history of HK, antiviral therapy should be applied before and for at least one year after the surgery [7].

In our previous study, we found viral DNA in 18.7% of recipients’ corneas undergoing keratoplasty without a history of HK and hence recommend PCR of all excised corneas [8].

However, little is known about the possible transmission of HSV and VZV from the donor tissue to the recipient. Bacteriological and mycotic screening examinations are part of the standard procedure of the eye banks prior to transplantation. Nevertheless, virological screening is not a routine examination in most eye banks [9]. Therefore, possible colonization and transmission of herpes viruses through the donor cornea cannot be excluded with certainty. Until now, the risk of viral transmission via corneal donation was considered negligible, as low viral prevalence has been demonstrated in previous studies. Broniek et al. found viral DNA in only 1.8% of the examined donor corneas [10].

The aim of this study was to determine the prevalence of herpes and cytomegalovirus (CMV) DNA in donor corneas and to investigate whether the transfer of a cornea with herpes DNA negatively affects the postoperative outcome of the graft. Furthermore, we aimed to clarify whether donors whose corneas viral DNA was detected differed from donors without viral DNA.

## 2. Materials and Methods

### 2.1. Study Design

This retrospective cohort study was conducted at the Department of Ophthalmology of Johannes Gutenberg University Mainz, Germany. We analyzed data from all donors and recipients who underwent penetrating keratoplasty (PK) or Descemet membrane endothelial keratoplasty (DMEK) between September 2022 and March 2023.

Corneoscleral rims of all donor corneas as well as all corneas excised from the recipients were collected on the day of the surgery and underwent PCR examination to detect HSV-1, herpes simplex virus type 2 (HSV-2), VZV, and CMV DNA. The PCR assays were performed at the Department of Virology, University Medical Center of Johannes Gutenberg University in Mainz, Germany.

Virological testing of the donors was performed as an internal quality control. Virological and microbiological testing of the recipients, regardless of the preoperative diagnosis, is part of our clinical routine. The results of the PCR were known 2–3 days after the surgery.

### 2.2. Preparation and Virological Testing

**Figure 1:**
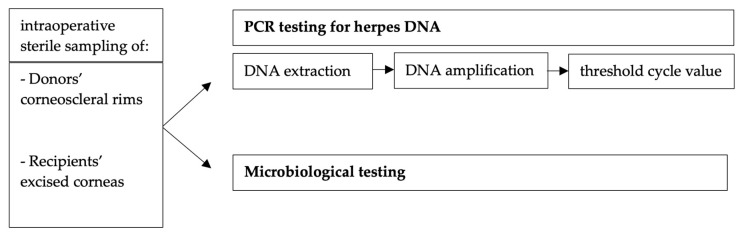
Procedures performed from sample collection to PCR result as part of our clinical routine and quality control.

We cut off tissue fragments measuring about 16 mm^2^ from the corneoscleral rims of all donor corneas as well as from all corneas excised from the recipients with sterile scissors, which were separate for each fragment. We sent these fragments for PCR testing. Herpes viral DNA was detected by PCR using a non-laboratory-developed real-time PCR test. DNA was extracted from corneal tissue using the AltoStar^®^ Automation System AM16 (Altona Diagnostics GmbH, Hamburg, Germany) according to the manufacturer’s instructions. Herpes viral DNA was amplified with the Bio-Rad CFX™ Real-Time PCR Detection System using primers provided by Altona Diagnostics PCR kits. The other fragments of the material were microbiologically tested according to our clinical routine (Figure 1).

### 2.3. Donors

Donor tissue was obtained from the Eye Bank of Rhineland-Palatinate, Mainz, Germany.

The donors’ characteristics, such as age, gender, lens status, endothelial cell density (ECD measured with REA (Robin Endothelial Analyzer, Robin GmbH, Haan, Germany), death-to-explantation interval (DEI), and cause of death (cardiovascular/cerebrovascular disease according to the International Classification of Diseases ICD-10 German Modification 17, chapter IX; cancer, sepsis), were documented according to the donor´s medical history and collected by an employee of the eye bank. The diagnosis of sepsis was made with evidence of systemic inflammatory response syndrome (SIRS) in association with clinically (blood gas analysis, blood count, infection-related organ dysfunction) or microbiologically proven infection. SIRS is present when ≥2 criteria of the following are met: a body temperature of ≥38 °C or ≤36 °C, hypocapnia (pCO_2_ ≤ 33 mmHg), breathing rate >20/min, heart rate >90/min, leucocytes 12.000/μL or <4.000/μL. In the case of external donors, the diagnosis was referred to the treating physicians.

At the eye bank, the tissue was stored at 34 °C in dextran-free cell culture medium (P04-09701 PanBiotech, Aidenbach, Germany) and at least 24 h before use transferred to 6% dextran-containing cell culture medium (P04-09702 PanBiotech, Aidenbach, Germany). Both media were supplemented with gamma-irradiated fetal calf serum, 2% or 10% in the case of donors younger than 40 years (S-FBS-AU-035, Serana, Pessin, Germany).

The cycle threshold (Ct) values indicate strong positive (<29 cycles of amplification), positive (30–40), or weak positive (>40) reactions with target nucleic acid.

### 2.4. Recipients

We collected the following recipients’ data: age, gender, ocular history (especially regarding previous ocular herpetic infections), and the type of corneal surgery.

After surgery, patients with a preoperative history of HK or with positive results for HSV-1, HSV-2, VZV, and CMV in the PCR of their excised cornea received topical and systemic antiviral treatment (acyclovir/ganciclovir ophthalmic ointment 1–5 times daily, acyclovir tablets 5 × 400 mg for the first 4 weeks, followed by 2 × 400 mg acyclovir for 1 year).

The decision regarding the application of the prophylactic antiviral treatment to the group of recipients of positively tested grafts was made individually in each case.

The recipients were followed up at regular intervals for up to one year and underwent slit lamp examination to detect symptoms of graft rejection, graft failure, or infection.

### 2.5. Statistics

In the first step, the collected data were described using descriptive statistics. The donor characteristics of the groups of positive and negative corneas were compared. A chi-square test was used to calculate any statistical relationship between two categorical characteristics. To test a continuous variable and a categorical variable for statistical significance, we used a *t*-test.

## 3. Results

### 3.1. Donors

We identified 107 donor corneoscleral rims, of which 23 had to be excluded due to missing information about the donor as these donor corneas were provided by external cornea banks. These also included one donor whose cornea tested positive for HSV-1. For further analysis, we included 85 corneas from 67 donors. Viral DNA was detected by means of PCR in 7 (8.24%) transplanted donor corneas, 4 of which were transferred by Descemet membrane endothelial keratoplasty (DMEK) and 3 by penetrating keratoplasty (PK). Three of the seven positively tested donor corneas (42.6%) were positive for HSV-1 DNA and four (57.1%) for VZV DNA. We did not detect any HSV-2 or CMV DNA. We present the characteristics of the donors in Table 1.

When examining the causes of death and previous diseases of corneal donors, we found sepsis in 3 of the 7 cases (42.9%) in the group of donors with corneas tested positive for herpetic viruses, compared to 21 of the 78 cases (26.9%) in the group of donors tested negative (*p* = 0.52). All 3 corneas from donors in a septic status at the time of death tested positive for VZV. In 6 of the septic patients, bloodstream infection was detected. We have not found any statistically significant difference in diagnoses, cultural duration, or DEI between positively and negatively tested donors.

### 3.2. Recipients

We included 85 eyes of 76 patients who received a corneal graft using PK or DMEK.

Seven eyes received donor corneas that tested positive for viral DNA, and 78 eyes received corneas that tested negative for viral DNA. We present the characteristics and postoperative follow-up of all recipients in Table 2.

In the postoperative follow-up, we noted relatively more surgical revisions in the recipients of positively tested grafts (42.7%) compared to recipients of negatively tested grafts (7.7%).

### 3.3. Postoperative Follow-Up of the Patients Who Received Corneal Grafts with Viral DNA

We present the follow-up of the patients who received the virus-positive donor corneas in Table 3. None of them had a history of viral disease, and no viral DNA was detectable in their own excised corneas. Prophylactic antiviral therapy was initiated in three of these patients to minimize the risk of donor-to-host transmission (#2, #3, #5). We observed graft failure in one (14.3%) case (#4). Postoperative infection/ulcer was observed in two (28.58%) cases (#2, #5). Three eyes (42.9%) needed surgical intervention, including one re-DMEK, one re-PK, and one anterior chamber revision. The remaining four recipients with evidence of herpes DNA in the corneal graft showed no signs of graft rejection, graft failure, or infection during the follow-up.

**Figure 2:**
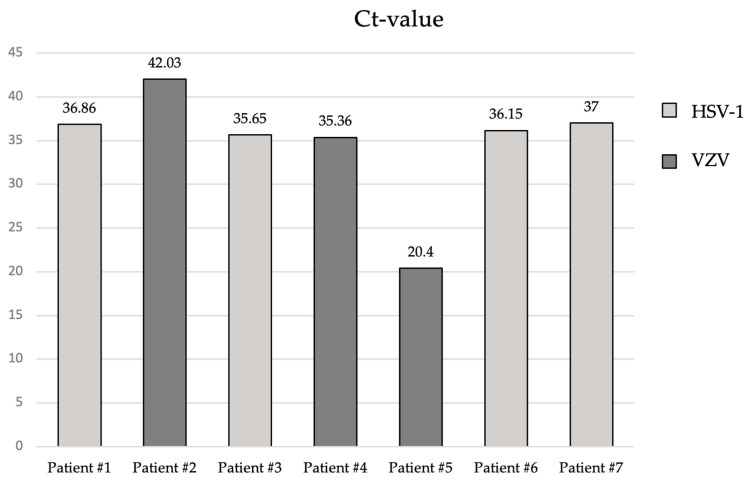
Cycle threshold (Ct)-values of herpes viral DNA from corneal grafts tested positive for HSV-1 and VZV DNA that patients #1-#7 received.

We present the Ct-values of the positively tested corneas in Figure 2. The Ct-values ranged from 20.4 to 42.03 and the mean Ct value was 34.82 ±/− 6.23.

### 3.4. Postoperative Follow-Up of the Patients Who Received Corneal Grafts with Viral DNA and Required Surgical Intervention during the Postoperative Follow-Up

#### 3.4.1. #4(FECD)

In this case, DMEK performed with the herpes-positive graft was complicated due to an intraoperative rise in intraocular pressure. We performed an anterior chamber deepening procedure and graft repositioning on the first postoperative day. Despite the immediate postoperative initiation of topical immunosuppressive therapy, a repeat DMEK was necessary 4 weeks postoperatively due to graft failure. A test of the explanted cornea was negative for the herpes virus. At 6-month follow-up, the cornea was clear and the transplant functioned very well.

#### 3.4.2. #2(Acanthamoebic Keratitis)

In this case, the initial PK with the herpes-positive graft had taken place due to massive contact-lens-associated acanthamoebic keratitis. Despite immediate postoperative initiation of topical immunosuppressive, antibacterial, antiacanthamoebic, and antiviral therapy, we observed a cloudy, Seidel-positive graft 12 weeks postoperatively. We performed a new PK and continued the local and systemic therapy. A test of the explanted cornea was negative for the herpes virus. At a 3-month follow-up, the corneal graft was clear and showed no signs of infection or rejection, but at a 6-month follow-up, the patient showed a massive intraocular infection with loosening of sutures, requiring a pars plana vitrectomy.

#### 3.4.3. #5(Ulcer in Corneal Graft)

We performed PK à chaud with a herpes-positive graft due to a perforated ulcer from a previous corneal transplant. Postoperatively, immediate initiation of immunosuppressive and systemic antiviral therapy could not prevent a renewed anterior chamber irritation as well as a thread loosening of the transplant three weeks after the PK à chaud. We performed an anterior chamber lavage and refixation of the sutures. Six months after PK, the patient’s corneal graft had remained clear.

## 4. Discussion

The aim of our study was to identify the prevalence of viral genomes in donor corneas and to evaluate the clinical follow-up of patients who received positively tested grafts. Previous studies showed a low prevalence of viral DNA in donor corneas [11]. Hence, the examination of donor corneas for viral DNA by PCR is not part of the standard infection diagnostics of the donated tissue. So far, the donor corneas have undergone microbiological testing to exclude bacterial or fungal contamination. Furthermore, donors’ blood is tested to identify hepatitis B virus (HBV), hepatitis C virus (HCV), syphilis, and human immunodeficiency virus (HIV) [10].

Graft-to-host transmission of HSV, especially type 1, has been described in some cases. Remeijer et al. provided conclusive evidence for the transmission of HSV-1 by penetrating keratoplasty with subsequent reactivation of donor-derived HSV-1 in the transplanted cornea. They found identical DNA sequences in the donor and recipient [12]. Reactivation of a latent infection in the graft may lead to a ‘newly acquired’ herpetic keratitis with persisting epithelial defects or even cause graft failure [13,14]. However, the clinical impact of graft-to-host herpetic infection is controversial because few individual cases of confirmed graft-to-host transmission have been diagnosed to date, and there is also no reliable data on the frequency of this rare phenomenon [11].

In a monocentric cohort study, Remeijer et al. analyzed 273 post-PK donor corneoscleral rims. They found HSV-1 DNA in 2 of 273 corneoscleral rims, leading to a prevalence of less than 1%. The authors therefore saw no diagnostic value in the virological screening of donors’ corneas regarding graft-to-host transmission or predicting the development of newly acquired HK after PK [11].

Comparable rates were shown in the study by Jing-Hao Qo et al. They found viral DNA in donor corneas in 2.44% of cases. Additionally, 0.64% of these tested positive for VZV DNA, 0.74% for HSV-1 DNA, 0.85% tested positive for CMV DNA, and 0.21% for EBV DNA. HSV-2 DNA was not detected by any of these authors [15]. Shimomura et al. showed comparably higher rates of 5.7% of herpes simplex DNA in donor corneas. This incidence was explicitly related to donors without a history of HK. In donors with a known history of HK, the authors detected herpes in the corneoscleral rims in as many as 10.8% of cases [16].

In this retrospective study, we analyzed the results of our internal quality control. We performed HSV-1, HSV-2, VZV, and CMV-PCR on all donor corneoscleral rims of the corneas transferred to patients by PK or DMEK between September 2022 and March 2023. Furthermore, according to our clinical routine, all excised recipients’ corneas underwent virological PCR tests [8].

We identified HSV-1 DNA in 3 grafts and VZV DNA in 4 grafts. We did not identify any HSV-2 or CMV DNA. The overall prevalence of human herpesvirus DNA in the donor cornea tissues was 8.24%, which is much higher than assumed and detected in the previous studies.

The source of the differences cannot be proven with certainty. Real-time PCR was used as the detection method in all studies. Moreover, in the Netherlands, cornea donors undergo serologic screening for hepatitis B virus and hepatitis C virus, and donor corneas are commonly cultured to detect bacterial and fungal infections. The ophthalmologic history of the donor is not taken as a standard prior to donation, either in Germany or in the Netherlands [11].

The sample size was smaller in our study (85 donor corneas vs. 273 corneoscleral rims in the study of Remejier), which therefore does not explain the higher rate of positive corneas.

One explanation could be the difference in the mean age of the corneal donors. In the study of Qo et al., the majority (54.5%) of the donors with positively tested corneas were between 30 and 50 years old, and 27.3% of the donors were between 51 and 61 years old. In our study, the mean age of donors of positively tested corneas was 67 years. Among donors with negatively tested corneas, the mean age was 79 in our study, and in Qo et al.’s study, the largest proportion (40%) of donors with negative corneas were between 30 and 50 years old. Thus, both groups in the study from Qo et al. tended to be younger compared with our study. Assuming that older donors also tended to have more comorbidities and were more likely to be hospitalized, this could explain the tendency for higher rates of herpes DNA in the donor corneas in our study. Remejier et al. have not specified the donors’ age in their study.

Biswas et al. have worked out why the transmission of viral DNA through corneal donation can pose a potential risk to the recipient. Graft-to-host transmission of herpes viruses can lead to primary graft failure and ulcerative keratitis after transplantation. They reported on endothelial destruction and necrosis of keratocytes in organ culture, therefore advocating the implementation of HSV-1 screening of donor corneas before PK [12,17].

In our study, three (42.9%) of the seven patients who received corneal grafts that tested positive for herpes DNA required reoperation with either re-PK, re-DMEK, or anterior chamber rinsing. In the group of negatively tested corneas, only 7.7% of patients required reoperation. However, in none of these cases with positively tested donor corneas, the herpetic genesis of the complications, such as infection or graft failure, could be determined with certainty. Instead, recurrence of the initial inflammation was suspected, as in the cases of acanthamoebic keratitis or pre-existing corneal ulcer. Particularly in the case of the third patient who required surgical revision, the graft failure was most likely a result of the initial complicated DMEK. The remaining four patients with detected herpes virus DNA in the corneal graft did not show any signs of infection, graft failure, or immune rejection.

In the group of patients who received positively tested corneal grafts in this study, none of the patients had a positive history of viral disease, and no viral DNA was detectable in their own excised corneas. This increased the probability that a postoperative detection of viral DNA in the donor corneas and a possible reactivation of HK were caused by graft-to-host transmission.

However, a reactivation of the herpes virus in the transplanted cornea could not be detected in any case, neither by PCR smear nor by sampling after explantation of the corneal graft. In two of the cases, postoperative antiviral therapy was initiated, which may have reduced the sensitivity of the PCR detection of viral DNA postoperatively. However, in the third case, no antiviral therapy was initiated, and no viral DNA was detected.

In general, a positive history of HK or latent viral infection does not always lead to a positive result in the virus-PCR, since the amount of viral DNA in the cornea steadily decreases after active inflammation [18]. Furthermore, Remeijer et al. showed in a prospective study that the viral load in corneas after HK is correlated with a fulminant disease and with potential steroid treatment of the recipient [11]. In this study, postoperative topical steroid treatment was initiated in all patients after PK or DMEK to prevent postoperative immune reactions. Thus, reactivation of herpes DNA cannot be excluded with certainty as a cause of graft failure in these cases since a detection might have been attenuated.

Upon differentiation of the viral DNA detected in this study, it became apparent that only VZV and HSV-1 DNA were detected. Three (42.7%) of the corneas tested positive for HSV-1 DNA, and four (57.1%) of them tested positive for VZV DNA. HSV-2 and CMV DNA were not detected in any case. However, the lack of detection of HSV-2 DNA in corneal tissue in this study is most likely a result of the different anatomical locations of latent HSV-2 compared with HSV-1 and VZV [19]. The higher rate of VZV-DNA compared to HSV-1 DNA contradicts the expected results, with VZV showing a relatively low reactivation frequency compared with HSV in immunocompetent individuals [20].

The Ct-values of PCR of the donor corneas ranged from 20.40 to 42.03. These values indicate strong positive (<29 cycles of amplification) reactions with a high amount of target nucleic acid and positive reactions (30–40) with moderate amounts of target nucleic acid. In only one case must a weak positive detection be assumed (>40). The recipient of this cornea had no postoperative complications and a satisfactory postoperative course. These Ct-values are comparable to Toth et al.’s retrospective study, in which they examined 206 corneal buttons without a history of HK for herpes simplex virus type 1 DNA by PCR. The average Ct value was 33.3, which was similar to the average Ct-value of our corneal transplants [21].

Despite the difficulty in detecting viral DNA in the postoperative patients with positive donor corneas, their ophthalmologic history and lack of clinical symptoms for HK suggest that the reoperations were most likely not caused by reactivation of the herpes virus, but by reactivation of initial bacterial colonization or complicated surgery. Nevertheless, we found the rate of postoperative complications high (3 of 7 patients) in comparison to our clinical experience and a relatively low reoperation rate in the group with herpes-negative grafts. Hence, we cannot exclude a possible correlation between viral last in the transplanted graft and postoperative complications. We hypothesize that corneal grafts with viral presence may be more susceptible to bacterial infections or less resistant to intraoperative stress.

Subsequently, this study aimed to clarify whether donors in whose corneal tissues viral DNA was detected differed from donors without viral detection and to investigate whether any of the donor characteristics increased the risk of herpes DNA in the cornea. For this purpose, we examined whether donors with viral DNA in the cornea were more likely to have sepsis at the time of death. In addition, we compared factors such as duration of corneal culture, lens status, cause of death, and time between death and tissue collection between the groups. The rate of sepsis was relatively high in the positively tested group compared to the negatively tested group (43% vs. 27%, respectively), but this effect was not statistically significant. In Germany, medical reasons for the exclusion of tissue donors include sepsis with multi-resistant bacterial pathogens (MRSA, VISA, VRSA, ESBL) or fungal sepsis. These pathogens are ruled out using blood sampling as part of the donor selection process. However, other causes of sepsis do not constitute a contraindication for tissue donation [22].

Furthermore, no statistically significant difference could be shown for any of the factors mentioned between donors with and without viral DNA. However, it must be noted that the statistical results were only of limited use due to the comparatively small group of donors who tested positive in the PCR.

Jing-hao Qu et al. suggest in a monocentric clinical study that the corneas of donors who had cancer, donors who were inpatients, and donors who had immunodeficiency or who were on immunosuppressive therapy had an increased risk of postsurgical endophthalmitis [15]. The authors suggest that the higher rate might have been caused by microbial transmission from donor to host and therefore recommend testing corneas from these patients also for herpes virus DNA. Moreover, some authors clearly argue for the implementation of viral screening of the excised corneas of recipients with a positive history of HK, especially on HSV-1.

At this point, the benefit of standardized screening of all corneas before transplantation is questionable, but herpes viral PCR of the donor cornea before transplantation may improve the postoperative outcome after PK or DMEK in individual cases. Especially in multimorbid donors who were hospitalized or suffering from sepsis at the time of death, an association with higher rates of herpes viral DNA and a negative effect on the postoperative outcome after transplantation has been pre-described and implicates that a screening of the cornea for herpes DNA may be useful [15]. Although not statistically significant, in this study, herpes DNA was detected more frequently in the cornea of donors with sepsis at the time of death than in patients without sepsis (43% vs. 27%, respectively).

However, medical factors such as scarcity of resources and donor corneas must also be taken into account, when assessing whether the benefit of regular PCR screening of every donor cornea offsets the expense. Regular screening of all donor corneas for viral DNA may improve postoperative outcomes in single, individual cases, but the cost-benefit ratio is questionable in view of the limited resources, donor numbers, and the unclear influence of transmission on the recipient.

## 5. Conclusions

We found human herpesvirus DNA in 8.24% of corneal grafts using PCR. The prevalence in this study was surprisingly higher than previously assumed. The reactivation of herpes viruses in the corneal graft is a high-risk factor for recurrent infection or graft failure. It was therefore important to investigate whether viral DNA in donor corneas negatively affects the postoperative outcome after transplantation. We observed a six-fold higher rate of postoperative complications/reoperations in the group of patients who received positively tested corneas. However, patients who received grafts with herpetic DNA and had postoperative complications, including surgical revisions did not show any clinical signs of HK or any evidence of herpetic DNA in the explanted tissue. Thus, we did not assume the donor-to-host transmission to be the cause of reoperation in any of these cases. However, we cannot exclude the possible influence of the virus on the postoperative condition of the tissue. We hypothesize that corneal grafts with viral presence may be more prone to bacterial infections or less resistant to intraoperative stress.

Due to the surprisingly high detection rate of herpes DNA in donor corneal tissues in this study, it is of high importance to further investigate, with a sufficiently large number of cases, whether the transfer of donor corneas with herpes viral DNA actually affects the postoperative outcome of the graft. The question of the necessity of virological testing in the eye banks remains open.

## Figures and Tables

**Table 1:** Characteristics of corneal donors in the virus-positive and virus-negative groups.

	Donor Corneas (n = 85)			*p*-Value (chi^2^)	*p*-Value (*t*-Test)
Herpes DNA in donor corneas (PCR)	positive n = 7	negative n = 78	total		
	7 (8.24%)	78 (91.76%)	85 (100%)		
Surgery					
DMEK	4 (57.14%)	54 (69.23%)	58	0.768003	
PK	3 (42.86%)	24 (30.76%)	27	0.648015	
Pseudophakia of donor	4 (57.14%)	52 (66.67%)		0.812914	
Mean age ± SD of donor [y]	69.29 ± 13.97	70.91 ± 10.68	70.77 ± 10.89		0.35
Range [years]	50–78	38–68	38–78		
Donor´s gender					
Male	2 (28.57%)	33 (42.31%)	35	0.633669	
Female	5 (71.42%)	45 (57.69%)	50	0.727894	
Cause of death					
Cancer	3 (42.86%)	26 (33.34%)	29	0.728771	
Systemic infection	3 (42.86%)	17 (21.79%)	20	0.353786	
Cardiovascular	1 (14.29%)	34 (43.59%)	35	0.283035	
Missing	0	1 (1.28%)	1	0.764623	
Sepsis	3 (42.86%)	21 (26.92%)	24	0.522728	
Cultural duration [hours]	15.86	18.01			0.12
Death-to-explantation interval [hours]	29.83	34.88			0.24

**Table 2:** Clinical information of the recipients. PK—penetrating keratoplasty, DMEK—Descemet membrane endothelial keratoplasty.

Characteristics	Recipients of Virus-Positive Donor Corneas N = 7	Recipients of Virus-Negative Donor Corneas N = 78
Mean age ± SD (range) of recipients [years]	67.43 ± 15.69 (37–81)	79.08 ± 15.02 (20–94)
Male/female	3/4	33/45
Diagnosis		
Fuchs endothelial corneal dystrophy	4	40
corneal ulcer/infection	3	5
other	0	33
Viral DNA in donor´s cornea	7	0
VZV	4 (4.71%)	0
HSV-1	3 (3.53%)	0
HSV-2	0	0
CMV	0	0
Viral DNA in recipient´s cornea	0	3
HSV-1	0	3
Surgery		
DMEK	4	54
PK	3	24
Surgical revision during follow-up	3 (42.86%)	6 (7.69%)

**Table 3:** Follow-up of patients who received the virus-positive donor corneas, ‘#’representing the number of the patient, ‘0’ representing no evidence of infection or immune rejection, ‘1’ representing evidence of infection, and ‘2’ graft failure. FECD—Fuchs endothelial corneal dystrophy, Ct-value—threshold cycle value; PK—penetrating keratoplasty, Re-PK—repeat penetrating keratoplasty, ACR—anterior chamber rinsing, Re-DMEK—repeat- Descemet membrane endothelial keratoplasty, PPV—pars plana vitrectomy, m—month.

Patients	#1	#2	#3	#4	#5	#6	#7
Initial reason for surgery	Graft failure	Acanthamoebic keratitis	FECD	FECD	Ulcer in the graft	FECD	FECD
Virus	VZV	HSV-1	VZV	HSV-1	HSV-1	VZV	VZV
Ct-value	36.86	42.03	35.65	35.36	20.40	36.15	37.00
Prophylactic antiviral treatment	-	+	+	-	+	-	-
Signs of infection or immune rejection at follow-up							
1 [m]	0	-	0	2 (Re-DMEK)	1 (ACR)	0	0
2 [m]	-	-	0	-	0	0	0
3 [m]	0	1 (Re-PK)	0	0	0	0	0
4 [m]	-	0	-	0	0	-	-
5 [m]	-	0	-	0	0	-	-
6 [m]	-	1 (PPV)	0	0	0	0	-
7 [m]	-	0	-	-		-	-

## Data Availability

Data are available on request.
